# Author Correction: Probing the limitations of multimodal language models for chemistry and materials research

**DOI:** 10.1038/s43588-025-00869-8

**Published:** 2025-08-21

**Authors:** Nawaf Alampara, Mara Schilling-Wilhelmi, Martiño Ríos-García, Indrajeet Mandal, Pranav Khetarpal, Hargun Singh Grover, N. M. Anoop Krishnan, Kevin Maik Jablonka

**Affiliations:** 1 Laboratory of Organic and Macromolecular Chemistry (IOMC), Friedrich Schiller University Jena, Jena, Germany; 2 School of Interdisciplinary Research, Indian Institute of Technology Delhi, Hauz Khas, New Delhi, India; 3 Department of Civil Engineering, Indian Institute of Technology Delhi, Hauz Khas, New Delhi, India; 4 Yardi School of Artificial Intelligence, Indian Institute of Technology Delhi, Hauz Khas, New Delhi, India; 5 Center for Energy and Environmental Chemistry Jena (CEEC Jena), Friedrich Schiller University Jena, Jena, Germany; 6 Helmholtz Institute for Polymers in Energy Applications Jena (HIPOLE Jena), Jena, Germany; 7 Jena Center for Soft Matter (JCSM), Friedrich Schiller University Jena, Jena, Germany

**Keywords:** Materials science, Chemistry

Correction to: *Nature Computational Science* 10.1038/s43588-025-00836-3, published online 11 August 2025.

In the version of the article initially published, the colours in the Fig. 3a key were reversed and have now been corrected in the HTML and PDF versions of the article, as seen in Fig. 1.

**Fig. 1:**
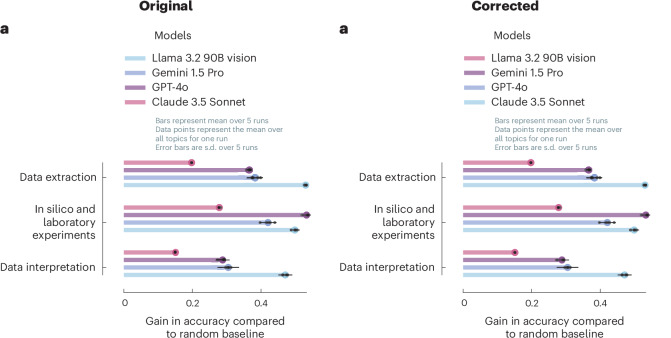
Original and corrected Fig. 3a.

