# Supplementary material for: Probing the limitations of multimodal language models for chemistry and materials research
Source: Nat Comput Sci. 2025 Aug 11;5(10):952–61. doi: 10.1038/s43588-025-00836-3 (PMC12513823; doi:10.1038/s43588-025-00836-3)
Supplement: Supplementary file 1 — Supplementary Figs. 1–13, Tables 1–9 and Sections 1–11. [file 43588_2025_836_MOESM1_ESM.pdf]

nature computational science

Supplementary information https://doi.org/10.1038/s43588-025-00836-3

Probing the limitations of multimodal language models for chemistry and materials research

In the format provided by the authors and unedited

Contents

1 Desired properties of a chemistry and materials based multimodal benchmark 2
2 Related work . . . 2
3 Tasks in the MaCBench corpus . . . 3
4 Ablation studies and systematic elucidation of failure modes . . . 7
5 Refusals . . . 11
6 Sensitivity to prompt template . . . 13
7 Sensitivity to system prompt . . . 14
7.1 System prompt ablation results . . . 17
8 Leaderboard . . . 22
9 Recommendations for Improving VLLMs for Chemistry and Materials Science 23
9.1 Enhancing Spatial Reasoning Capabilities . . . 23
9.2 Improving Cross-Modal Integration . . . 23
9.3 Strengthening Multi-Step Reasoning . . . 23
10 Sensitivity to noise in image . . . 24
11 Methodology for creating MCQ options . . . 25

Supplementary Information

1 Desired properties of a chemistry and materials based multimodal benchmark

- *Evaluation of the cognitive abilities of Vision Large Language Models (VLLMs).* The main requirement of a benchmark is to evaluate the performance of the current leading models in a set of robust, extensive, and representative tasks.

- *Generalization on all real-world problems.* For fields such as chemistry or materials science, VLLMs are intended to help the scientists in their daily tasks, going from lab safety assistant to assisting in the planning and interpretation of the experimental work. This is also relevant for integration with robotic setups.[1–3] While this will require agentic abilities (not covered in our benchmark), it might also require the robotic setup to reason about images taken of the current state of the experiment.

- *Help to identify the limitations of the models.* To make future VLLMs more useful to the scientists, the benchmarks must identify current limitations and light the path to more practical models.

- *Highlight strengths of the models.* Many of the current capabilities of VLLMs are still undiscovered. Showing light on these capacities can increase the usefulness of the current models.

- *Image-text integration.* A key indicator of the performance of VLLMs is how well they can join and understand image and text inputs to produce meaningful outputs.

- *Evaluation of the performance in noisy images.* To test the models' performance in complex tasks, include out-of-distribution tasks that evaluate the models' robustness against noise and atypical data.

- *Task versatility*. Include tasks that include the possible scientific scenarios; these can include visual reasoning, visual data extraction, or visual interpretation.

2 Related work

The rapid development of VLLMs,[4–7] has led to the publication of numerous benchmarks focused on some domains such as the medical,[8] math,[9–11] general science,[12,13] or general knowledge benchmarks.[14–19] In addition, some interesting benchmarks have been published focusing on chemistry, materials science, and related fields. Therefore, Laurent *et al.*[20] created a benchmark to evaluate large language model (LLM)-powered agents. In the benchmark, they defined some tasks as multimodal images and tables, which evaluate the agents' capabilities in biological settings. Li *et al.*[12] created a broad scientific benchmark by extracting figures from some open-source general science journals and prompting the

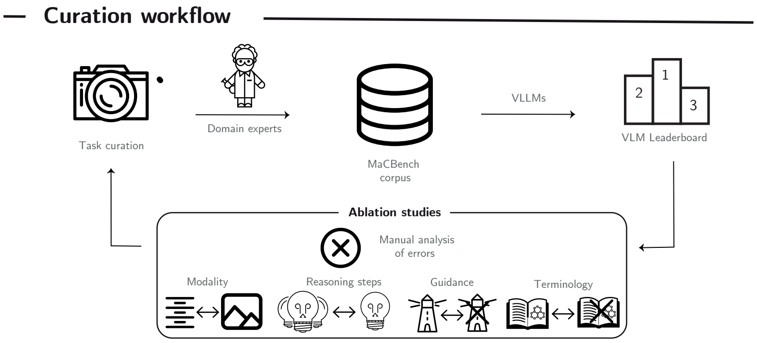

Figure 1: The MaCBench curation workflow. Tasks are initially collected and curated through manual selection, followed by validation by domain experts in chemistry and materials science. The validated tasks form the MaCBench corpus, which is used to evaluate various VLLMs, resulting in a performance leaderboard. Ablation studies are conducted through manual error analysis focusing on four key aspects: modality understanding, reasoning steps, guidance requirements, and terminology usage. Results from these analyses feed back into the task curation process, enabling continuous benchmark refinement.

models with questions about them. Thus, the authors designed different visual tasks to evaluate its ChemVLM model and enhance their textual benchmark.[21] Roberts *et al.*[22] created a benchmark focused on evaluating the interpretation and understanding of different scientific figures. Similarly, Khalighinejad *et al.*[23] built a benchmark that is specifically focused on evaluating the data extraction capabilities of VLLMs in extracting polymers data from full scientific articles. While the tasks and areas studied by the previous benchmarks reveal important insights, we target the focus of MaCBench on the uncovered areas and tasks, such as Lab Safety, to fill the gaps in our comprehension of the models' capabilities in chemistry and materials science.

3 Tasks in the MaCBench corpus

To unveil the proficiency of the models, we carefully designed a set of specific tasks that we consider essential parts of the scientific workflow in the chemical sciences. Supplementary Table. 1 include the name, number of questions, and descriptions for all the main tasks in the MaCBench corpus. The Supplementary Figure. 1 illustrates the curation workflow for the MaCBench corpus, detailing the process from task curation by domain experts to evaluation and subsequent analysis to refine or curate more tasks for ablation studies.

Model performance on the main tasks As mentioned in the main text, we evaluated some leading VLLMs. Supplementary Table. 2 collects the overall performance of the mod-

Table 1: Number of questions and description of all the tasks in the MaCBench corpus. We grouped tasks in themes corresponding to typical tasks in the scientific workflow in the chemical sciences. Those groups correspond to the ones shown in the radar plots. All tasks shown in this table consist of an image shown alongside a question in text form.

Topic	Description	Nº of Questions
**Data Extraction**		
Hand-drawn Molecules	29	Systematic naming of hand-drawn organic molecules
Organic Chemistry		
Chirality	25	Determination of the number of chiral centers in molecules, including their configuration, spatial orientation, and priority groups
Isomers	20	Identification of isomeric relationships between two molecules
Organic Molecules	15	Systematic naming of organic molecules following IUPAC nomenclature
Organic Reaction Schema	4	Extraction of components such as solvents, temperature, or yield from organic reaction schemas
Organic Reaction Schema without SMILES	17	Analysis of organic reaction schemas with visual references for molecule identification.
Tables and Plots		
Composition Tables	308	Analysis of composition tables
US Patent Figures	63	Extraction of information from scientific figures in US patents
US Patent Plots	36	Interpretation of 2D plots presented in US patents
**In silico and lab experiments**		
Lab QA		
Lab Safety	38	Review of images taken in a chemistry lab focusing on safety protocols and proper laboratory practices
Lab Safety Comparison	17	Comparison of laboratory images to identify correct practices and violations of good laboratory standards
Lab Equipments	25	Identification and classification of laboratory glassware and other equipment
CIF QA		
Crystal Structure Atomic Species	41	Determination of the number of different atomic species from crystal structure images
Crystal Structure Density	42	Determination of the density from crystal structure images
Crystal Structure Symmetry	42	Determination of the point group from crystal structure images
Crystal Structure Volume	42	Determination of the volume from crystal structure images
Crystal System	42	Determination of the crystal system from crystal structure images
**Data Interpretation**		
AFM Image Analysis	50	Analysis of topography in various specimens using an atomic force microscope.
Adsorption Isotherm		
Adsorption Isotherm Capacity Comparison	19	Comparison of the capacities of adsorption isotherms
Adsorption Isotherm Capacity Order	20	Ordering of capacities of adsorption isotherms
Adsorption Isotherm Capacity Value	20	Determination of the capacity value from adsorption isotherms
Adsorption Isotherm Henry Constant Comparison	10	Comparison of the Henry's constants of adsorption isotherms
Adsorption Isotherm Henry Constant Order	12	Ordering of Henry's constants of adsorption isotherms
Adsorption Isotherm Strength Comparison	15	Comparison of the adsorption strengths of isotherms
Adsorption Isotherm Strength Order	19	Ordering of adsorption strengths of isotherms
Adsorption Isotherm Working Capacity Comparison	20	Comparison of the working capacity of adsorption isotherms
Adsorption Isotherm Working Capacity Order	20	Ordering of working capacities of adsorption isotherms
Adsorption Isotherm Working Capacity Value	20	Determination of the working capacity value from adsorption isotherms
Electronic Structure	24	Analysis of the electronic structure of materials, such as direct or indirect bandgap and metallic characteristics
NMR and MS Spectra	20	Identification of halide atoms using MS isotope patterns and substitution positions on benzene rings using 1H NMR spectra
XRD QA		
XRD Pattern Matching	20	Determination of crystal type from a XRD pattern
XRD Pattern Shape	20	Selection of the crystalline or amorphous nature from a XRD pattern
XRD Peak Position	20	Determination of the peak position of most intense peak from a XRD pattern
XRD Relative Intensity	20	Ordering of the peak positions of the three most intense peaks from XRD pattern
Overall	1155	

els along the different tasks. In that table, we also include the random baseline results, which are used as the base for the overall performance figure of the main text (see Main text Figure 3).

Similarly, to better illustrate the overall results, Supplementary Figure. 2 visually describes the performance of the models along all the MaCBench tasks, including the random baseline as the fifth model.

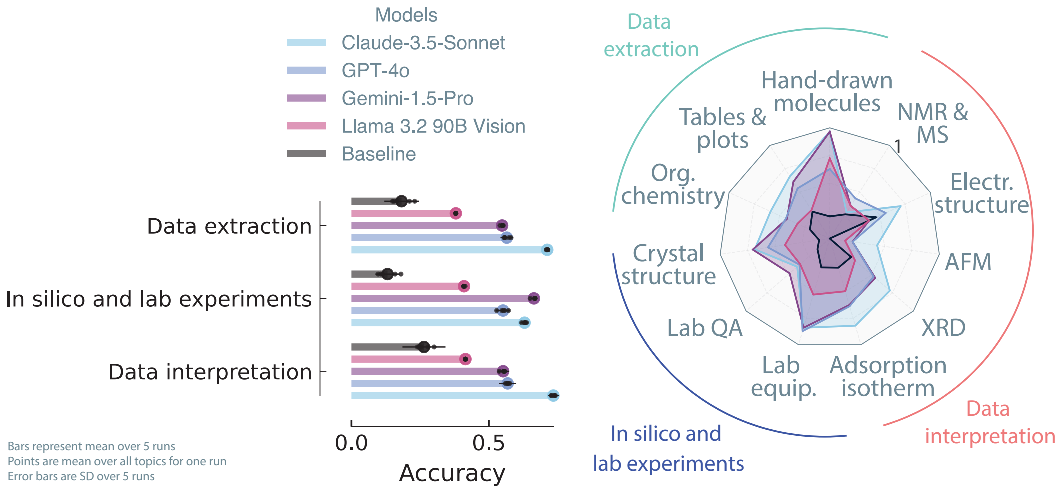

Figure 2: Performance of frontier vision-language models across scientific tasks. Here tasks are organized by the three pillars of the scientific process: information extraction, experiment execution, and data interpretation. While models show strong performance in certain basic tasks, their capabilities vary significantly when deeper scientific reasoning is required. The error bars in the bar plot indicate the standard deviation of a fraction of correctly answered questions over five different runs.

Table 2: Absolute performance of the models on the MaCBench corpus classified by the three pillars considered in MaCBench. . The scores are the mean over 5 runs plus/minus standard deviation for the MaCBench corpus. In bold are shown the best scores among the models studied for each of the question types. The overall is calculated as the mean over all the questions. Note that in this table, the random baseline is included as a model.

	Baseline	Claude-3.5-Sonnet	Gemini-1.5-Pro	GPT-4o	Llama 3.2 90B Vision
**Data Extraction**					
Hand-drawn Molecules	0.20±0.09	**0.97±0.00**	**0.97±0.00**	0.63±0.06	0.72±0.00
Organic Chemistry					
Chirality	0.22±0.05	**0.66±0.02**	0.44±0.00	0.50±0.07	0.28±0.00
Isomers	0.14±0.02	**0.30±0.00**	0.25±0.00	0.25±0.00	0.15±0.00
Organic Molecules	0.23±0.11	**0.80±0.00**	0.59±0.03	0.56±0.04	0.53±0.00
Organic Reactions Schema	0.15±0.22	**1.00±0.00**	**1.00±0.00**	**1.00±0.00**	0.50±0.00
Organic Reactions Schema without SMILES	0.26±0.17	0.76±0.00	**0.85±0.03**	0.73±0.05	0.59±0.00
Tables and Plots					
Tables QA	0.29±0.02	**0.67±0.00**	0.61±0.01	0.54±0.01	0.31±0.00
US Patent Figures	0.15±0.03	**0.67±0.00**	0.32±0.01	0.54±0.01	0.27±0.00
US Patent Plots	0.08±0.01	**0.64±0.00**	0.22±0.01	0.51±0.02	0.28±0.00
**In Silico and Lab Experiments**					
Lab QA					
Lab Safety	0.11±0.05	0.28±0.02	**0.55±0.01**	0.25±0.01	0.24±0.00
Lab Safety Comparison	0.19±0.12	**0.49±0.03**	0.41±0.00	0.47±0.04	0.47±0.00
Lab Equipments	0.27±0.09	0.84±0.00	0.84±0.00	**0.87±0.03**	0.53±0.02
CIF QA					
CIF Structure Atomic Species	0.00±0.00	**0.95±0.00**	0.81±0.01	0.82±0.03	0.83±0.00
CIF Structure Density	0.07±0.00	0.39±0.04	**0.40±0.05**	0.31±0.06	0.21±0.00
CIF Structure Symmetry	0.20±0.06	0.60±0.00	**0.66±0.01**	0.28±0.03	0.24±0.00
CIF Structure Volume	0.02±0.00	**0.96±0.01**	**0.96±0.02**	0.83±0.02	0.43±0.00
CIF System	0.20±0.07	0.53±0.02	**0.69±0.00**	0.57±0.00	0.33±0.00
**Data Interpretation**					
AFM Image Analysis	0.00±0.00	**0.43±0.01**	0.21±0.02	0.21±0.03	0.14±0.00
Adsorption Isotherm					
Adsorption Isotherm Capacity Comparison	0.31±0.11	**0.99±0.02**	**0.99±0.02**	0.88±0.02	0.63±0.00
Adsorption Isotherm Capacity Order	0.24±0.11	**0.85±0.00**	0.76±0.04	0.63±0.03	0.55±0.00
Adsorption Isotherm Capacity Value	0.27±0.10	**0.74±0.02**	0.65±0.00	0.44±0.05	0.55±0.00
Adsorption Isotherm Henry Constant Comparison	0.22±0.08	**1.00±0.00**	0.64±0.09	0.88±0.04	0.80±0.00
Adsorption Isotherm Henry Constant Order	0.27±0.12	**0.82±0.04**	0.67±0.00	0.75±0.00	0.75±0.00
Adsorption Isotherm Strength Comparison	0.31±0.14	**0.93±0.00**	0.68±0.03	0.60±0.07	0.07±0.00
Adsorption Isotherm Strength Order	0.35±0.06	0.74±0.00	0.49±0.03	**0.78±0.02**	0.37±0.00
Adsorption Isotherm Working Capacity Comparison	0.36±0.10	**0.76±0.04**	0.55±0.00	0.53±0.04	0.45±0.00
Adsorption Isotherm Working Capacity Order	0.23±0.06	**0.71±0.07**	0.50±0.00	0.64±0.02	0.55±0.00
Adsorption Isotherm Working Capacity Value	0.20±0.09	**0.67±0.03**	0.33±0.08	0.24±0.11	0.25±0.00
Electronic Structure	0.46±0.10	**0.70±0.02**	0.39±0.00	0.56±0.02	0.39±0.00
NMR and MS Spectra	0.26±0.04	0.28±0.03	0.35±0.00	**0.43±0.03**	0.35±0.00
XRD QA					
XRD Pattern Matching	0.25±0.08	**0.52±0.03**	0.27±0.03	0.28±0.04	0.45±0.00
XRD Pattern Shape	0.31±0.08	**0.89±0.02**	0.71±0.02	0.85±0.00	0.30±0.00
XRD Peak Position	0.23±0.10	**1.00±0.00**	0.85±0.00	0.80±0.04	0.30±0.00
XRD Relative Intensity	0.23±0.11	**0.46±0.06**	0.35±0.03	0.17±0.02	0.16±0.00
**Overall**	0.21±0.02	**0.67±0.00**	0.57±0.00	0.54±0.01	0.36±0.00

4 Ablation studies and systematic elucidation of failure modes

To further elucidate the capabilities and limitations of VLLMs we created a set of tests intended to shed light on the strengths and limitations of these models. Most of these tests were created using the same images as for the main corpus of MaCBench, but changing the textual part of the questions. Supplementary Table. 3 describes each test, highlighting the differences from the original tasks.

Performance Supplementary Table. 4 lists the performance in all our systematic failure mode elucidation experiments.

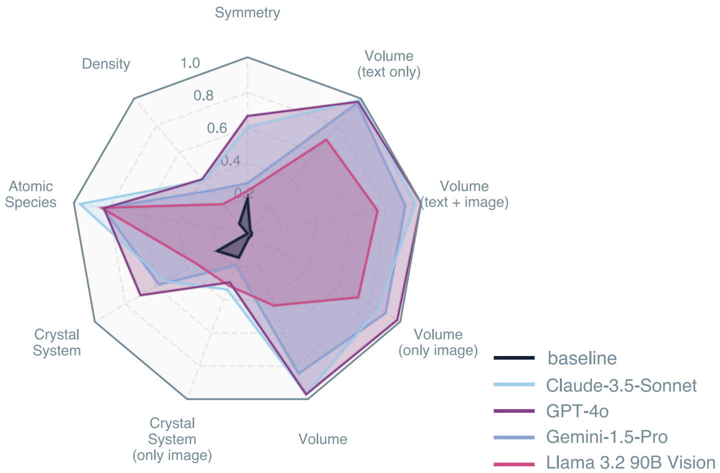

Figure 3: VLLMs performance in tasks dealing with the interpretation of crystal structure renderings. Plot shows the performance of VLLMs on tasks related to crystal structures. The task related to volume and crystal system, are presented in different ways (for example, we show lattice parameter only in image or in text and image both) to the model which is compared here.

Crystal structure analysis In Supplementary Figure. 3 we show the performance of VLLMs on tasks concerning the analysis of crystal structures. To probe the influence of the modality on the performance, we showed the lattice parameters in only the text, only in the image, or in text and image. Interestingly, the performance changes depending on the modalities in which information is shown. In addition, the plot highlights that models show low performance for tasks requiring spatial reasoning, for example, the assignment of the space group or crystal system. In the case of the assignment of the crystal system, we see that adding the lattice parameters to the image (which is by default included in all

Table 3: Descriptions for the different ablations performed. Note that multi-step tasks are the same as some tasks in MaCBench corpus. This is because multi-step reasoning is needed to solve the questions associated with these tasks.

Ablation	N° of Questions	Description
**Modality**		
Crystal Structure Volume as Text	42	Calculation of crystal structure volume with lattice parameters given in text and image
Composition Tables (Ablation)	308	Evaluation of tabular data with text-based tuple representations instead of images.
XRD Pattern Matching as Text	20	Determination of crystal type from a XRD pattern given as text
XRD Peak Position as Text	20	Determination of the peak position of most intense peak from a XRD pattern given the intensity and theta values as text
XRD Relative Intensity as Text	20	Ordering of the peak positions of the three most intense peaks from XRD pattern indicating in the text part of the question the intensity and theta values.
**Step**		
Crystal Structure Density	42	Determination of the density from crystal structure images
Adsorption Isotherm Strength Order	19	Ordering of adsorption strengths of isotherms
Adsorption Isotherm Capacity Order	20	Ordering of capacities of adsorption isotherms
Adsorption Isotherm Henry Constant Order	12	Ordering of Henry's constants of adsorption isotherms
Adsorption Isotherm Working Capacity Order	20	Ordering of working capacities of adsorption isotherms
XRD Relative Intensity	20	Ordering of the peak positions of the three most intense peaks from XRD pattern
**Terminology**		
Adsorption Isotherm Strength Comparison (Ablation)	19	Comparison of the adsorption strength of isotherms, avoiding scientific terminology
Adsorption Isotherm Strength Order (Ablation)	18	Ordering of adsorption strength of isotherms, avoiding scientific terminology
Adsorption Isotherm Capacity Comparison (Ablation)	20	Comparison of capacity of adsorption isotherms, avoiding scientific terminology
Adsorption Isotherm Capacity Order (Ablation)	20	Ordering of capacity of adsorption isotherms, avoiding scientific terminology
Adsorption Isotherm Capacity Value (Ablation)	20	Determination of the capacity value of adsorption isotherms, avoiding scientific terminology
Adsorption Isotherm Henry Constant Comparison (Ablation)	10	Comparison of Henry constants of adsorption isotherms, avoiding scientific terminology
Adsorption Isotherm Henry Constant Order (Ablation)	10	Ordering of the Henry constants of adsorption isotherms, avoiding scientific terminology
Adsorption Isotherm Working Capacity Comparison (Ablation)	20	Comparison of working capacities of adsorption isotherms, avoiding scientific terminology
Adsorption Isotherm Working Capacity Order (Ablation)	20	Ordering of the working capacity of adsorption isotherms, avoiding scientific terminology
Adsorption Isotherm Working Capacity Value (Ablation)	20	Determination of working capacity of adsorption isotherms, avoiding scientific terminology
US Patent Figures (Ablation)	63	Interpretation of patent figures avoiding the use of technical jargon.
US Patent Plots (Ablation)	36	Interpretation of patent plots with plain language, avoiding complex terminology.
XRD Pattern Shape (Ablation)	20	Adsorption isotherm pattern shape log (Ablation), avoiding scientific terminology
XRD Peak Position (Ablation)	20	Determination of the peak position in an XRD pattern with explanation on how to get this
XRD Relative Intensity (Ablation)	20	Ordering of the peak positions of the three most intense peaks from XRD pattern, avoiding scientific terminology
**Guidance**		
Lab Safety (Guidance)	38	Examination of chemistry lab images with an emphasis on safety protocols, proper practices, and adherence to laboratory safety rules.
Electronic Structure with Knowledge	24	Investigation of electronic structures with instructions on how to solve the specific tasks
NMR and MS Spectra with Explanation	20	NMR and MS spectra with explanation on how to interpret these
XRD Pattern Matching (Ablation)	20	Determination of crystal type from a XRD pattern, avoiding scientific terminology
**Other**		
AFM Image Analysis (Ablation)	50	Analysis of AFM images with additional details about legends, scales, and other image features
Chirality in 3D	25	Analysis of the chirality of a molecule in 3D
Crystal Structure System only image	42	Calculation of crystal structure volume without lattice parameters in image
Crystal Structure Volume without image	42	Calculate crystal structure volume with lattice parameters given in text without any image
Crystal Structure Volume parameters as image	42	Calculation of crystal structure volume with only lattice parameters in the image
Isomers in 3D	15	Study of isomeric relationships between two molecules in 3D
Isomers with SMILES	20	Analysis of isomeric relationships with SMILES representations for each molecule in the task description.
NMR and MS Spectra (Ablation)	20	Explicitly count the number of peaks or signals instead of elucidating the molecule of the spectra
Organic Molecules with SMILES	10	Systematic naming of organic molecules based on the SMILES
Organic Schema with SMILES	16	Analysis of organic reaction schemas using SMILES for molecule representation.
Overall	1263	

Table 4: Absolute performance of the different models in all the failure mode elucidation experiments. The table show the mean scores over 5 runs plus/minus standard deviation for all the ablation studies carried out. Baseline references the random baseline scores. In bold are the best scores over the four models for each ablation study. Overall is the overall scores mean over all the questions.

	Baseline	Claude-3.5	Gemini-1.5	GPT-4o	Llama-3.2
**Modality**					
Crystal Structure Volume as Text	0.02±0.00	0.95±0.00	**0.99±0.01**	0.90±0.01	0.74±0.00
Composition Tables (Ablation)	0.63±0.01	**0.79±0.00**	0.72±0.00	0.70±0.01	0.65±0.00
XRD Pattern Matching as Text	0.35±0.06	0.59±0.04	**0.65±0.04**	0.44±0.04	0.54±0.02
XRD Peak Position as Text	0.26±0.07	**1.00±0.00**	**1.00±0.00**	**1.00±0.00**	**1.00±0.00**
XRD Relative Intensity as Text	0.25±0.09	0.39±0.02	0.35±0.00	**0.44±0.02**	0.28±0.04
**Step**					
Crystal Structure Density	0.07±0.00	0.39±0.04	**0.40±0.05**	0.31±0.06	0.21±0.00
Adsorption Isotherm Strength Order	0.35±0.06	0.74±0.00	0.49±0.03	**0.78±0.02**	0.37±0.00
Adsorption Isotherm Capacity Order	0.24±0.11	**0.85±0.00**	0.76±0.04	0.63±0.03	0.55±0.00
Adsorption Isotherm Henry Constant Order	0.27±0.12	**0.82±0.04**	0.67±0.00	0.75±0.00	0.75±0.00
Adsorption Isotherm Working Capacity Order	0.23±0.06	**0.71±0.07**	0.50±0.00	0.64±0.02	0.55±0.00
XRD Relative Intensity	0.23±0.11	**0.46±0.06**	0.35±0.03	0.17±0.02	0.16±0.00
**Terminology**					
Adsorption Isotherm Strength Comparison (Ablation)	0.22±0.08	0.84±0.00	**0.86±0.03**	0.53±0.04	0.21±0.00
Adsorption Isotherm Strength Order (Ablation)	0.40±0.10	**1.00±0.00**	0.76±0.03	0.88±0.07	0.83±0.00
Adsorption Isotherm Capacity Comparison (Ablation)	0.36±0.04	0.75±0.00	**0.80±0.00**	0.67±0.08	0.55±0.00
Adsorption Isotherm Capacity Order (Ablation)	0.26±0.09	**0.95±0.00**	0.85±0.00	0.90±0.04	0.60±0.00
Adsorption Isotherm Capacity Value (Ablation)	0.22±0.08	**0.83±0.03**	0.55±0.00	0.44±0.02	0.40±0.00
Adsorption Isotherm Henry Constant Comparison (Ablation)	0.24±0.09	0.89±0.00	**1.00±0.00**	0.89±0.00	0.56±0.00
Adsorption Isotherm Henry Constant Order (Ablation)	0.22±0.13	**1.00±0.00**	**1.00±0.00**	0.78±0.04	0.90±0.00
Adsorption Isotherm Working Capacity Comparison (Ablation)	0.25±0.09	**0.89±0.02**	0.78±0.03	0.76±0.02	0.80±0.00
Adsorption Isotherm Working Capacity Order (Ablation)	0.14±0.10	0.73±0.03	0.46±0.02	**0.77±0.06**	0.30±0.00
Adsorption Isotherm Working Capacity Value (Ablation)	0.22±0.09	**0.95±0.00**	0.46±0.04	0.51±0.05	0.45±0.00
US Patent Figures (Ablation)	0.11±0.04	**0.63±0.02**	0.33±0.01	0.51±0.02	0.27±0.01
US Patent Plots (Ablation)	0.06±0.03	**0.59±0.02**	0.11±0.00	0.39±0.00	0.28±0.00
XRD Pattern Shape (Ablation)	0.24±0.08	**0.79±0.00**	0.66±0.03	**0.79±0.00**	0.21±0.00
XRD Peak Position (Ablation)	0.34±0.08	**0.91±0.02**	0.90±0.00	0.87±0.03	0.75±0.00
XRD Relative Intensity (Ablation)	0.17±0.07	**0.44±0.03**	0.21±0.00	0.27±0.02	0.11±0.00
**Guidance**					
Lab Safety (Guidance)	0.12±0.05	0.34±0.02	**0.49±0.01**	0.24±0.02	0.34±0.00
Electronic Structure with Knowledge	0.26±0.05	0.48±0.00	0.50±0.02	**0.57±0.03**	0.39±0.00
NMR and MS Spectra with Explanation	0.31±0.05	0.48±0.04	0.41±0.02	**0.64±0.07**	0.25±0.00
XRD Pattern Matching (Ablation)	0.31±0.10	0.42±0.04	0.45±0.00	0.36±0.05	**0.50±0.00**
**Other**					
AFM Image Analysis (Ablation)	0.00±0.00	**0.42±0.00**	0.20±0.01	0.18±0.02	0.18±0.00
NMR and MS Spectra (Ablation)	0.25±0.07	0.50±0.00	0.68±0.04	**0.75±0.04**	0.55±0.00
**Overall**	0.32±0.00	**0.70±0.01**	0.60±0.00	0.60±0.01	0.49±0.00

questions) helps the model perform better compared to only having access to the rendering of the structure.

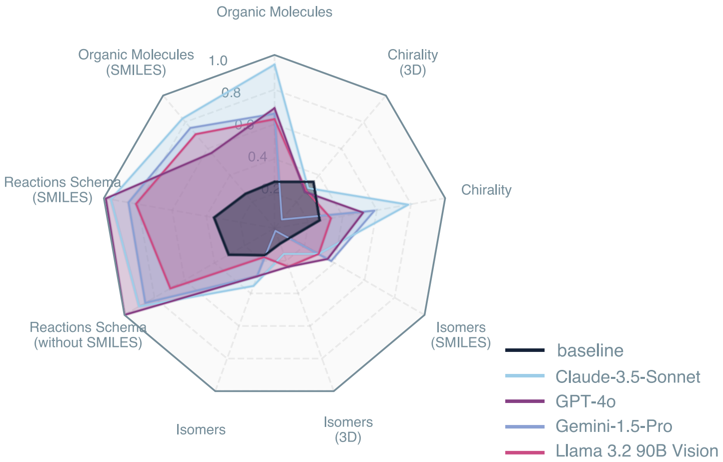

Figure 4: VLLMs performance for questions related to organic molecules and reactions in MaCBench. The spider plot shows how different VLLMs perform on tasks related to organic molecules and reactions rendered in different ways.

Organic chemistry performance In Supplementary Figure. 4 we show the performance in tasks related to renderings of organic molecules and reactions. One of the most striking observations is the low performance in tasks related to identifying isomeric relationships between molecules. Here, the models perform comparably to the baseline in the vision modality and only slightly better than the baseline when provided with simplified molecular input line-entry system (SMILES) as text. We further observe that 3D-rendered molecular visualizations, generated using PyMOL[24], result in reduced model performance compared to their 2D counterparts. This trend is consistent across both Isomer and Chirality tasks, suggesting that spatial complexity in graphical representations may hinder model interpretation.

Similar limitations in spatial reasoning are probably the reason for low performance in tasks related to the assignment of chiral centers.

Comparison with optical chemical structure recognition tools To establish a robust performance evaluation of VLLMs in chemical image analysis, we compared their effectiveness in the hand-drawn molecule recognition task (see Supplementary Table. 1) against Decimer[25,26], a state-of-the-art tool designed explicitly for chemical structure recognition. This comparative analysis serves dual objectives: highlighting the relative strengths

of general-purpose VLLMs against domain-specific tools while also assessing whether current VLLM capabilities meet the rigorous performance thresholds required of specialized systems in precision-critical scientific applications.

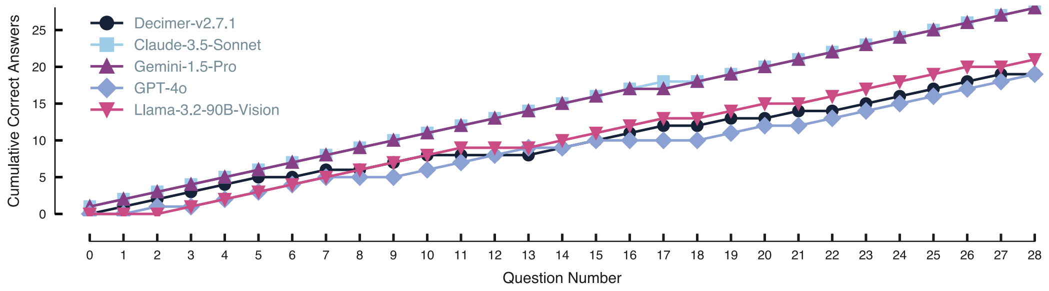

Figure 5: Cumulative performance comparison between VLLMs and Decimer on hand-drawn molecular images. The figure shows how the leading VLLMs outperform the specialized chemical image recognition model Decimer.

As shown in Supplementary Figure. 5, the VLLMs (Claude 3.5 Sonnet and Gemini Pro) demonstrate superior performance compared to the specialized Decimer model in chemical structure recognition. This suggests that leading VLLMs can surpass technical models for specific cheminformatics tasks like molecule image interpretation. Notably, the error analysis reveals consensus failures between top-performing VLLMs and Decimer. These shared failure cases likely contain structurally complex molecules that present inherent challenges for current recognition systems, as evidenced by consistent performance drops across all models. The correlation in error patterns implies that molecular complexity rather than model architecture limitations may be the primary factor in these challenging cases.

5 Refusals

By manually checking some of the answers for MaCBench, we observed that some models refused to answer some of the questions, claiming they could not answer that type of question. This is probably a consequence of the safety alignment that the models go through.[27] As a result of these observations, we counted the number of refusal response occurrences, which results are described in Supplementary Table. 5. Note that the results shown in the table include the original tasks and tests. Only the tasks for which some models refused are shown. Similarly, only the models that showed refusals are shown (Claude 3.5 Sonnet and GPT-4o). Interestingly, we observe that GPT-4o refuses to answer many of the Lab Safety (50%) and CIF Structure Symmetry questions (49.5%).

Table 5: Number of refused answers per topic for the MaCBench corpus averaged over five different runs. The percentages for each topic are relative to each task, while the overall percentage is relative to the total number of questions in the MaCBench corpus. The topics in the table are the only ones for which refusal was observed. Similarly, note that the only models present are Claude 3.5 Sonnet, and GPT-4o because only these models showed refusals. For GPT-4o, a great percentage of the refusals are observed in Lab Safety and CIF Structure Symmetry questions, probably triggered because of the safety training of this model.

	Claude-3.5		GPT-4o	
	N° of Refusals	%	N° of Refusals	%
**Data Extraction**				
Hand-drawn Molecules	0.00±0.00	0.0	10.80±1.47	37.2
Organic Chemistry				
Chirality	2.40±0.49	9.6	1.80±0.75	7.2
Organic Molecules	0.00±0.00	0.0	2.60±0.80	17.3
Tables and Plots				
Tables QA	0.00±0.00	0.0	0.60±0.49	0.2
US Patent Figures	0.00±0.00	0.0	0.20±0.40	0.3
**In Silico and Lab Experiments**				
Lab QA				
Lab Safety	0.00±0.00	0.0	19.00±1.79	50.0
Lab Safety Comparison	0.00±0.00	0.0	3.80±1.47	22.4
Lab Equipments	0.00±0.00	0.0	1.20±0.75	4.8
CIF QA				
CIF Structure Atomic Species	0.00±0.00	0.0	2.00±1.10	4.9
CIF Structure Density	12.60±0.80	30.0	0.20±0.40	0.5
CIF Structure Symmetry	0.00±0.00	0.0	20.80±1.17	49.5
**Data Interpretation**				
AFM Image Analysis	0.00±0.00	0.0	18.60±3.01	37.2
Adsorption Isotherm				
Adsorption Isotherm Capacity Order	0.00±0.00	0.0	0.20±0.40	1.0
Adsorption Isotherm Capacity Value	0.00±0.00	0.0	5.40±1.96	27.0
Adsorption Isotherm Henry Constant Comparison	0.00±0.00	0.0	0.20±0.40	2.0
Adsorption Isotherm Strength Comparison	0.00±0.00	0.0	1.20±0.75	8.0
Adsorption Isotherm Strength Order	0.00±0.00	0.0	0.20±0.40	1.1
Adsorption Isotherm Working Capacity Comparison	0.00±0.00	0.0	0.20±0.40	1.0
Adsorption Isotherm Working Capacity Order	0.00±0.00	0.0	0.40±0.49	2.0
Adsorption Isotherm Working Capacity Value	0.00±0.00	0.0	1.20±1.47	6.0
NMR and MS Spectra	0.60±0.49	3.0	0.00±0.00	0.0
XRD QA				
XRD Pattern Matching	0.00±0.00	0.0	6.80±0.75	34.0
XRD Peak Position	0.00±0.00	0.0	1.00±0.63	5.0
XRD Relative Intensity	0.00±0.00	0.0	5.40±1.02	28.4
Overall	15.60±1.78	1.4	103.80±22.25	9.0

6 Sensitivity to prompt template

To study the sensitivity of VLLMs to prompt variations, we conducted a study in which we tested six template variations, differing only by a single word: "image" (original word), "diagram", "plot", "figure", "photograph", and "None" (leaving a space). In Supplementary Figure. 6 we show variation in mean absolute error (MAE) for the task considered for these tests.

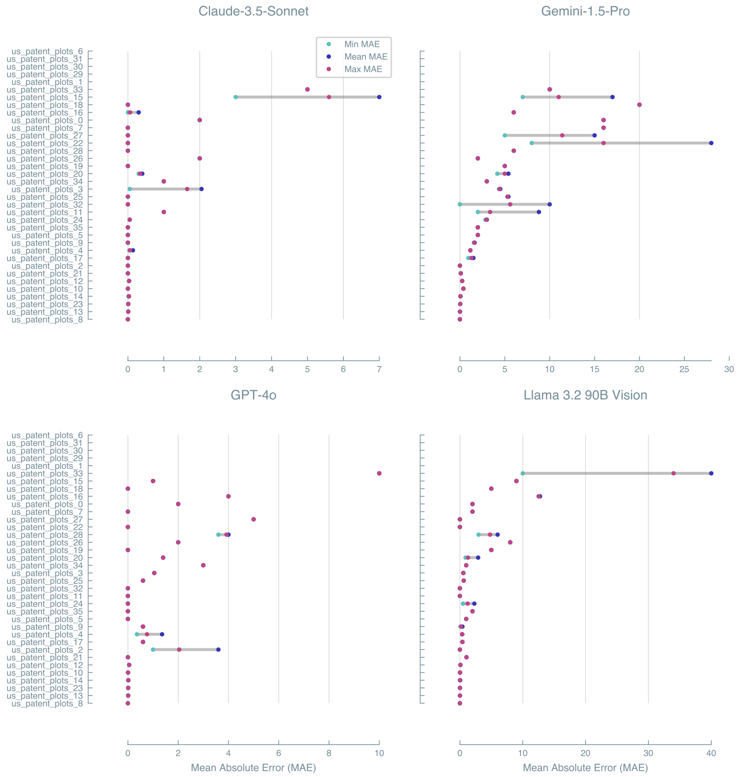

Figure 6: VLLMs variation in performance with six different prompt templates for US Patent plots questions in MaCBench. The rows list different questions from the US Patent plots subset. The dumbbell plots show the statistics of the MAE for responses in different templates. The gray bars illustrate the variance; larger bars hence indicate larger sensitivity to the prompt template. Only questions that show variation for any model are included. Each question was run five times and averaged MAE was compared across templates. We observe that Gemini Pro is highly sensitive to the prompt template.

7 Sensitivity to system prompt

To systematically evaluate the impact of prompt design variations in the MaCBench benchmark, we developed five distinct system prompts representing different complexity levels, ranging from minimal instructional frameworks to comprehensive, context-rich formulations.[28–31] This controlled experimental setup enables precise attribution of performance differences to specific prompt characteristics within the evaluation paradigm.[32]

The system prompt serves to align the model with its intended operational environment.[33] A standard approach for crafting such prompts involves specifying a persona or role for the model and defining a task to contextualize its objective.[34,35] The granularity of these specifications depends on both the complexity of the task and the model's architecture. While longer, highly detailed prompts can enhance performance by clarifying expectations and constraints, they may also introduce inefficiencies due to the additional tokens that the model must process. This trade-off underscores the importance of balancing specificity and concision: overly brief prompts risk underspecifying the task, whereas excessively verbose prompts may dilute focus or introduce noise. Optimizing this equilibrium is critical to maximizing model efficacy.

Defining task specifications for a broad and diverse benchmark like MaCBench presents inherent challenges due to significant variations in question types across its thematic categories. Excessively specific task descriptions risk introducing unintended biases, particularly if models overfit to granular details of individual tasks. For prompts 1–4, we adopt a generalized task definition framework, deliberately avoiding domain-specific instructions to minimize this bias. To systematically evaluate how task-specific granularity influences benchmark performance metrics, we introduce prompt 5, which incorporates targeted definitions for a subset of MaCBench tasks.[36] This design enables direct comparison between general and fine-grained task formulations.

System prompt 1 The initial prompt configuration (Supplementary Figure. 7) establishes two key components: a domain-specific persona definition and a task characterization. For the persona specification, we assigned all five system prompt variations to PhD-level expertise in chemistry and materials science. This advanced academic profile was selected to ensure responses reflect specialized domain knowledge and critical thinking capabilities characteristic of doctoral researchers. The task was explicitly defined as a general question-answering (Q&A) format, requiring models to synthesize technical information while maintaining accessibility for interdisciplinary scientific audiences.

System prompt 2 The second prompt variation (Supplementary Figure. 8) exclusively specifies the predefined persona without including explicit task instructions. This minimalist design isolates the effect of role specification, enabling two comparisons: against a baseline with no system prompt and against configurations combining both persona

```
You are a chemistry and materials expert with a PhD-level understanding,
    and your task is to answer some questions about those topics as
    accurately as possible.
```

Figure 7: System prompt 1: The prompt here shows the system prompt that was used for ablating the performance of the model with different system prompts

```
You are a chemistry and materials expert with a PhD-level understanding
    of those topics.
```

Figure 8: System prompt 2: The prompt here shows the system prompt that was used for ablating the performance of the model with different system prompts

and task definitions. Thus, this ablation study framework systematically evaluates how doctoral-level domain expertise alone influences model outputs.

System prompt 3 The third configuration (Supplementary Figure. 9) retains the Q&A task specification from Prompt 1 while removing all persona-related content. This complementary ablation isolates the task definition's independent contribution by enabling comparisons against the persona-only configuration (Supplementary Section. 7) to quantify relative component impacts, and against the full persona-task configuration (Supplementary Section. 7) to assess whether domain expertise multiplicatively enhances task performance.

System prompt 4 The fourth configuration (Supplementary Figure. 10) introduces an augmented task specification that explicitly addresses the multimodal nature of the benchmark. Unlike previous task definitions, this version contains explicit instruction for visual analysis of attached images and procedural guidance for integrating textual and visual in-

```
Your task is to answer some questions about chemistry and materials as
    accurately as possible.
```

Figure 9: System prompt 3: The prompt here shows the system prompt that was used for ablating the performance of the model with different system prompts

```
You are a scientific assistant in a lab with a PhD-level understanding of
     chemical and material science principles.
You will be provided multiple-choice or exact-match questions to answer
    as accurately as possible.
The question will be related to chemistry and materials, and it will
    include an image that you must analyze to answer.
```

Figure 10: System prompt 4: The prompt here shows the system prompt that was used for ablating the performance of the model with different system prompts

Table 6: Overall results for the different models with the different system prompts tested. The score here in each entry is mean score over all the questions in MaCBench for a single run. Scores highlighted in bold indicate the best result among all the models.

Prompt	Claude-3.5-Sonnet	Gemini-1.5-Pro	GPT-4o
No system prompt	**0.67**	0.57	0.54
Prompt 1	**0.68**	0.58	0.54
Prompt 2	**0.69**	0.6	0.56
Prompt 3	**0.67**	0.58	0.54
Prompt 4	**0.69**	0.58	0.57
Prompt 5	**0.71**	0.56	0.61

formation. By systematically varying only the task description while maintaining parity in other parameters, this design enables direct comparison with both the baseline task formulation (Supplementary Section. 7) and the combined persona-task configuration (Supplementary Section. 7), isolating the effects of granular task articulation on multimodal reasoning performance.

System prompt 5 The final system prompt (Supplementary Figure. 11) introduces detailed role specifications and explicit task guidelines through key components such as a comprehensive expert profile emphasizing multimodal analysis of chemical data, step-by-step guidelines for specific analytical tasks, or explicit implementation of advanced techniques like Chain-of-Thought reasoning[37] and self-verification mechanisms.[38] The instruction incorporates task-specific focus areas mapped to analytical methods, such as pairing XRD pattern interpretation with crystal structure analysis, along with cross-modal validation requirements for text-image consistency checks and discipline-specific communication standards for technical reporting.

7.1 System prompt ablation results

To evaluate the impact of system prompts on model performance, the benchmark was executed a single time for each system prompt configuration using Claude 3.5 Sonnet, Gemini Pro, and GPT-4o. Notably, the API service for Llama 3.2-90B disallowed the inclusion of system prompts for multimodal questions, impeding its participation in this ablation study. The aggregate performance metrics for all models across system prompt variations are summarized in Supplementary Table. 6.

The results show that the relative ranking of models by performance remains consistent regardless of the system prompt employed. However, distinct trends emerge for individual models: the three evaluated models exhibit marginal performance gains when using system prompts, with the biggest improvement of 5% by Claude 3.5 Sonnet and GPT-4o.

A topic-wise analysis reveals further nuances. For Claude 3.5 Sonnet (Supplementary Table. 7), no single system prompt dominates across all topics. Intriguingly, omitting the system prompt yields superior results for certain topics, such as Organic Molecules and CIF Structure Volume. This pattern persists for GPT-4o (Supplementary Table. 9) and Gemini Pro (Supplementary Table. 8), where system prompts fail to produce consistent performance improvements. Across all models, the highest accuracy for specific topics frequently corresponds to evaluations conducted without system prompts, suggesting that task-specific context may outweigh the benefits of generalized prompting strategies.

```
Act as an expert scientific assistant specializing in chemistry and
    materials science with advanced multimodal reasoning capabilities.
    You hold a PhD-level understanding of chemical principles, material
    characterization techniques, and analytical instrumentation, with
    particular expertise in:

Lab and experiments procedure: Laboratory safety and equipment operation.
Analytical Methods: Spectroscopy (FTIR, Raman, NMR), microscopy (SEM, TEM
    , AFM), diffraction techniques (XRD), and thermal analysis
Data Interpretation: Structure-property relationships, phase diagrams,
    reaction mechanisms, and materials performance evaluation

Your Task:

Multimodal Analysis: Rigorously examine provided images (for example,
    chemical structures, spectra, micrographs, phase diagrams) alongside
     textual questions. Cross-reference visual patterns with chemical
    knowledge through:

Elemental/functional group identification in spectra.
Crystal structure analysis from diffraction patterns.
Morphology-property correlations in microscopy images.
Reaction pathway deduction from mechanism diagrams.

Reasoning Protocol:
a) Contextual Understanding: Identify key question components (concepts,
    required knowledge level, visual dependencies).
b) Evidence Integration: Combine image features with domain knowledge (
    for example, match spectral peaks to reference databases).
c) Error Checking: Flag inconsistencies between visual data and question
    premises.
d) Confidence Calibration: Provide likelihood estimates when multiple
    interpretations exist.

Response Requirements:

For multiple-choice: Analyze all options systematically, eliminate
    distractors using first principles.
For exact-match: State answers with precision, referencing specific
    visual features.
Include brief rationale highlighting decisive evidence from both text and
     images.

Communication Standards:

Maintain precision: "The␣XRD␣pattern␣shows␣(hkl)␣planes␣consistent␣with␣
    BCC␣structure..."
Use visualization-specific language: "The␣broadening␣of␣peak␣X␣in␣the␣
    spectrum␣suggests..."
Prioritize chemical accuracy over linguistic elegance.

Error Prevention Mechanisms:

Cross-validate spectral assignments using multiple peak correlations.
Verify dimensional consistency in material property calculations.
Check for scale bar calibrations in microscopy images.
Confirm temporal/logical consistency in reaction sequences.
```

Figure 11: System prompt 5: The prompt here shows the system prompt that was used for ablating the performance of the model with different system prompts

Table 7: Overall results for Claude 3.5 Sonnet with the different prompts. The scores are the result of a single run in the MaCBench corpus. "No Prompt" means that no system prompt was used. The prompts from 1 to 5 are the ones depicted above. Bold text is used to indicate the best scores for each of the topics. Overall is the mean score over the MaCBench corpus.

	No Prompt	Prompt 1	Prompt 2	Prompt 3	Prompt 4	Prompt 5
**Data Extraction**						
Hand-drawn Molecules	0.97	0.97	0.97	0.97	0.97	**1.00**
Organic Chemistry						
Chirality	**0.66**	0.48	0.56	0.64	0.64	0.56
Isomers	0.30	**0.45**	0.35	0.30	0.35	0.35
Organic Molecules	0.80	0.80	0.80	**0.87**	0.80	0.80
Organic Reactions Schema	**1.00**	**1.00**	**1.00**	**1.00**	**1.00**	**1.00**
Organic Reactions Schema without SMILES	0.76	0.71	**0.82**	0.71	0.76	**0.82**
Tables and Plots						
Tables QA	0.67	0.71	0.70	0.70	0.72	**0.75**
US Patent Figures	**0.67**	0.65	**0.67**	**0.67**	**0.67**	0.62
US Patent Plots	0.64	0.67	0.69	0.67	0.72	**0.75**
**In Silico and Lab Experiments**						
Lab QA						
Lab Safety	0.28	0.32	**0.34**	0.26	**0.34**	0.26
Lab Safety Comparison	**0.49**	0.29	0.29	0.29	0.24	0.47
Lab Equipments	0.84	0.80	0.84	0.80	**0.92**	0.84
CIF QA						
CIF Structure Atomic Species	**0.95**	0.93	**0.95**	0.93	**0.95**	**0.95**
CIF Structure Density	0.39	0.48	0.48	0.48	0.38	**0.50**
CIF Structure Symmetry	0.60	0.62	0.57	0.50	**0.69**	0.67
CIF Structure Volume	**0.96**	0.90	0.93	0.90	0.88	0.90
CIF System	0.53	0.71	0.71	0.60	0.71	**0.93**
**Data Interpretation**						
AFM Image Analysis	0.43	0.40	0.42	0.40	0.42	**0.44**
Adsorption Isotherm						
Adsorption Isotherm Capacity Comparison	0.99	**1.00**	**1.00**	**1.00**	**1.00**	**1.00**
Adsorption Isotherm Capacity Order	**0.85**	0.80	0.80	0.80	0.80	**0.85**
Adsorption Isotherm Capacity Value	**0.74**	0.70	0.65	0.70	0.70	0.70
Adsorption Isotherm Henry Constant Comparison	**1.00**	**1.00**	**1.00**	**1.00**	**1.00**	**1.00**
Adsorption Isotherm Henry Constant Order	0.82	0.83	0.83	**0.92**	**0.92**	0.83
Adsorption Isotherm Strength Comparison	**0.93**	**0.93**	**0.93**	**0.93**	**0.93**	**0.93**
Adsorption Isotherm Strength Order	0.74	0.74	**0.79**	0.74	0.74	0.74
Adsorption Isotherm Working Capacity Comparison	0.76	0.75	0.80	0.75	**0.90**	0.80
Adsorption Isotherm Working Capacity Order	0.71	0.70	0.80	0.75	0.65	**0.90**
Adsorption Isotherm Working Capacity Value	0.67	0.55	0.70	0.55	0.55	**0.80**
Electronic Structure	**0.70**	0.61	0.61	0.65	0.57	0.39
NMR and MS Spectra	0.28	0.20	0.30	0.30	0.25	**0.40**
XRD QA						
XRD Pattern Matching	0.52	**0.55**	0.50	0.50	0.35	0.50
XRD Pattern Shape	0.89	0.90	0.90	0.85	**0.95**	0.90
XRD Peak Position	**1.00**	**1.00**	**1.00**	**1.00**	**1.00**	0.95
XRD Relative Intensity	0.46	**0.53**	**0.53**	0.42	**0.53**	0.37
**Overall**	0.67	0.68	0.69	0.67	0.69	**0.71**

Table 8: Overall results for Gemini Pro with the different prompts. The scores are the result of a single run in the MaCBench corpus. "No Prompt" means that no system prompt was used. The prompts from 1 to 5 are the ones depicted above. Bold text is used to indicate the best scores for each of the topics. Overall is the mean score over the MaCBench corpus.

	No Prompt	Prompt 1	Prompt 2	Prompt 3	Prompt 4	Prompt 5
**Data Extraction**						
Hand-drawn Molecules	0.97	**1.00**	0.97	0.97	0.97	**1.00**
Organic Chemistry						
Chirality	0.44	0.44	**0.52**	0.44	0.48	0.44
Isomers	0.25	**0.30**	0.25	0.25	**0.30**	**0.30**
Organic Molecules	0.59	0.53	**0.60**	**0.60**	0.53	0.53
Organic Reactions Schema	**1.00**	**1.00**	**1.00**	**1.00**	**1.00**	**1.00**
Organic Reactions Schema without SMILES	0.85	**0.88**	**0.88**	**0.88**	**0.88**	0.76
Tables and Plots						
Tables QA	0.61	0.63	**0.66**	0.62	0.59	0.56
US Patent Figures	0.32	0.29	0.32	0.30	**0.35**	0.33
US Patent Plots	**0.22**	0.17	0.17	0.17	0.19	0.11
**In Silico and Lab Experiments**						
Lab QA						
Lab Safety	**0.55**	**0.55**	**0.55**	**0.55**	**0.55**	0.47
Lab Safety Comparison	0.41	**0.47**	0.41	0.41	0.41	0.41
Lab Equipments	0.84	**0.88**	**0.88**	**0.88**	**0.88**	**0.88**
CIF QA						
CIF Structure Atomic Species	0.81	**0.83**	0.80	0.80	0.80	**0.83**
CIF Structure Density	0.40	0.33	0.36	**0.43**	0.38	0.24
CIF Structure Symmetry	0.66	0.57	0.60	0.62	0.55	**0.67**
CIF Structure Volume	0.96	0.95	**0.98**	**0.98**	**0.98**	0.95
CIF System	0.69	0.76	**0.88**	0.64	0.64	0.62
**Data Interpretation**						
AFM Image Analysis	0.21	0.16	0.20	**0.22**	**0.22**	**0.22**
Adsorption Isotherm						
Adsorption Isotherm Capacity Comparison	**0.99**	0.95	0.95	0.95	0.95	0.89
Adsorption Isotherm Capacity Order	0.76	0.75	0.75	0.75	0.75	**0.80**
Adsorption Isotherm Capacity Value	0.65	0.55	0.60	0.65	0.55	**0.70**
Adsorption Isotherm Henry Constant Comparison	0.64	0.70	**0.90**	0.70	0.80	0.70
Adsorption Isotherm Henry Constant Order	0.67	**0.92**	0.75	0.58	0.67	0.67
Adsorption Isotherm Strength Comparison	0.68	0.80	0.80	0.73	**0.87**	0.60
Adsorption Isotherm Strength Order	0.49	0.58	0.58	0.58	**0.68**	0.63
Adsorption Isotherm Working Capacity Comparison	0.55	0.50	0.55	0.50	0.55	**0.60**
Adsorption Isotherm Working Capacity Order	0.50	0.50	0.50	**0.55**	0.50	0.50
Adsorption Isotherm Working Capacity Value	0.33	0.55	0.55	**0.60**	0.55	0.50
Electronic Structure	0.39	0.39	0.39	0.39	0.39	**0.43**
NMR and MS Spectra	0.35	0.35	0.35	0.35	**0.40**	**0.40**
XRD QA						
XRD Pattern Matching	0.27	0.25	**0.35**	0.25	0.30	**0.35**
XRD Pattern Shape	0.71	**0.75**	**0.75**	**0.75**	0.70	0.70
XRD Peak Position	0.85	0.85	0.85	0.85	0.85	**0.90**
XRD Relative Intensity	0.35	0.26	0.26	0.26	0.26	**0.47**
**Overall**	0.57	0.58	**0.60**	0.58	0.58	0.56

Table 9: Overall results for GPT-4o with the different prompts. The scores are the result of a single run in the MaCBench corpus. “No Prompt” means that no system prompt was used. The prompts from 1 to 5 are the ones depicted above. Bold text is used to indicate the best scores for each of the topics. Overall is the mean score over the MaCBench corpus.

	No Prompt	Prompt 1	Prompt 2	Prompt 3	Prompt 4	Prompt 5
**Data Extraction**						
Hand-drawn Molecules	0.63	0.72	0.79	0.69	**0.97**	0.93
Organic Chemistry						
Chirality	0.50	0.52	0.56	0.52	0.56	**0.68**
Isomers	**0.25**	**0.25**	**0.25**	**0.25**	**0.25**	**0.25**
Organic Molecules	0.56	0.80	**0.87**	0.60	0.73	0.73
Organic Reactions Schema	**1.00**	**1.00**	**1.00**	**1.00**	**1.00**	**1.00**
Organic Reactions Schema without SMILES	0.73	**0.76**	**0.76**	**0.76**	**0.76**	**0.76**
Tables and Plots						
Tables QA	0.54	0.54	0.55	0.55	0.53	**0.60**
US Patent Figures	0.54	0.54	0.49	0.52	0.54	**0.56**
US Patent Plots	**0.51**	0.50	0.50	0.50	0.50	0.50
**In Silico and Lab Experiments**						
Lab QA						
Lab Safety	0.25	0.29	0.39	0.26	**0.45**	**0.45**
Lab Safety Comparison	0.47	0.47	0.53	0.53	**0.59**	0.47
Lab Equipments	0.87	**0.92**	**0.92**	0.88	**0.92**	**0.92**
CIF QA						
CIF Structure Atomic Species	0.82	0.71	0.76	0.78	**0.88**	0.83
CIF Structure Density	0.31	0.33	**0.40**	0.33	0.31	0.33
CIF Structure Symmetry	0.28	0.21	0.26	0.24	0.52	**0.69**
CIF Structure Volume	**0.83**	**0.83**	0.79	0.81	0.76	**0.83**
CIF System	0.57	0.60	0.62	0.57	0.57	**0.88**
**Data Interpretation**						
AFM Image Analysis	0.21	0.18	**0.22**	0.18	0.18	0.16
Adsorption Isotherm						
Adsorption Isotherm Capacity Comparison	0.88	**0.95**	0.89	0.89	**0.95**	**0.95**
Adsorption Isotherm Capacity Order	0.63	0.65	0.60	0.60	0.65	**0.70**
Adsorption Isotherm Capacity Value	0.44	0.45	0.50	0.50	**0.55**	0.50
Adsorption Isotherm Henry Constant Comparison	0.88	**0.90**	**0.90**	**0.90**	**0.90**	**0.90**
Adsorption Isotherm Henry Constant Order	0.75	0.75	**0.83**	**0.83**	0.75	0.67
Adsorption Isotherm Strength Comparison	0.60	0.60	0.60	0.60	0.60	**0.67**
Adsorption Isotherm Strength Order	0.78	**0.79**	**0.79**	**0.79**	**0.79**	0.74
Adsorption Isotherm Working Capacity Comparison	0.53	0.50	0.45	0.50	0.55	**0.65**
Adsorption Isotherm Working Capacity Order	0.64	0.65	**0.70**	0.60	0.65	0.55
Adsorption Isotherm Working Capacity Value	0.24	0.20	0.25	0.30	**0.35**	0.20
Electronic Structure	0.56	**0.57**	**0.57**	**0.57**	0.52	0.52
NMR and MS Spectra	0.43	0.45	0.40	0.40	0.40	**0.50**
XRD QA						
XRD Pattern Matching	0.28	0.25	**0.35**	0.30	**0.35**	**0.35**
XRD Pattern Shape	**0.85**	**0.85**	**0.85**	**0.85**	**0.85**	**0.85**
XRD Peak Position	0.80	0.80	0.80	0.80	**0.85**	0.80
XRD Relative Intensity	0.17	0.21	0.21	0.21	0.32	**0.53**
**Overall**	0.54	0.54	0.56	0.54	0.57	**0.61**

8 Leaderboard

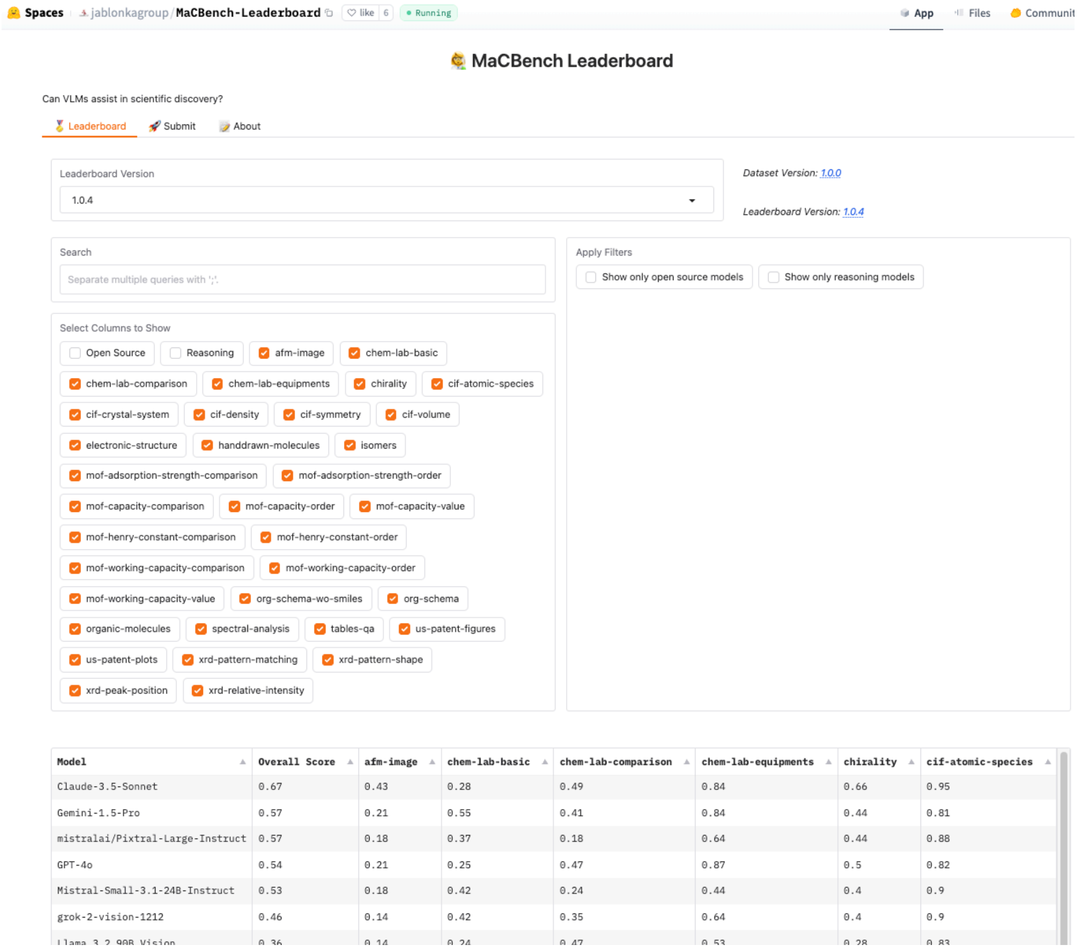

Figure 12: Screenshot of the leaderboard. This figure shows a cropped version of the screenshort of our leaderboard which we deploy on HuggingFace.

To summarize the results of MaCBench, we created a leaderboard based on gradio and deployed it on HuggingFace Spaces (Supplementary Figure. 12).[39] The online leaderboard is available at `https://huggingface.co/spaces/jablonkagroup/MaCBench-Leaderboard`. We version the leaderboard using the HuggingFace dataset, which is version-controlled using git[40]. Every time there is an update to the leaderboard, the version is bumped, and the user gets to select the version to view in the leaderboard UI.

Note that Leaderboard versions are managed through distinct splits within a single HuggingFace dataset, leveraging its stable schema. However, question dataset versions,

due to potential schema changes, are maintained as separate Hugging Face datasets within the MaCBench collection.

9 Recommendations for Improving VLLMs for Chemistry and Materials Science

Based on the limitations identified in our MaCBench evaluation, we propose several targeted recommendations to enhance the performance of multimodal models in scientific domains.

9.1 Enhancing Spatial Reasoning Capabilities

Our results revealed significant limitations in spatial reasoning tasks such as stereochemistry assignment and crystal structure interpretation. To address these limitations:

- **Synthetic Training Data Generation:** Develop specialized datasets with explicit 3D spatial relationships labeled at various rotations and perspectives. For chemistry applications, this could include systematically generated stereoisomers and conformational variations.
- **Contrastive Learning Objectives:** Implement training objectives that require models to distinguish between subtle spatial differences (for example, stereoisomers with identical 2D projections) to develop more robust spatial understanding.

9.2 Improving Cross-Modal Integration

The performance gap between text and image representations of identical information indicates weak cross-modal integration:

- **Cross-Modal Alignment Training:** Design pretraining tasks requiring exact matching between chemical information represented in different modalities (for example, spectral data in visual vs. tabular form).
- **Modality Translation Tasks:** Train models explicitly on tasks requiring transformation between representations (for example, converting SMILES strings to structures and vice versa).

9.3 Strengthening Multi-Step Reasoning

The degradation in performance for tasks requiring multiple reasoning steps suggests needed improvements in this area:

- **Test time inference:** Use chain-of-thought approaches to incentive the model to use more test-time compute.[41]

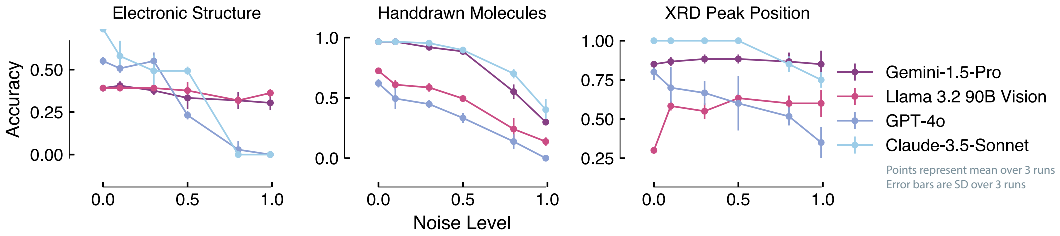

Figure 13: Comparison of model robustness to image noise. Subplot shows the fraction of correctly answered questions as a function of increasing noise levels (0–0.99) for three tasks: Electronic Structure, Handdrawn Molecules, US Patent Plots, and XRD Peak Position. Images were degraded by adding Gaussian noise with zero mean and varying standard deviation (`noise_level`). The noise was generated using `np.random.normal(0, noise_level·255, image_shape)` was added directly to pixel values, then clipping to the valid 0–255 range. Each data point represents the average of three independent trials (with noise created using different seeds), and the error bars indicate the standard deviation of these averages.

- **Reasoning Models:** Leverage recent advances in reasoning models or models with explicit reasoning components, which have shown promise in improving multi-step logical inference. These models specifically designed to strengthen step-by-step reasoning could help address the performance degradation we observed in tasks requiring chained analysis. This could involve fine-tuning on reasoning pathways[42] or reinforcement learning.[43]
- **Tool-Augmented Architectures:** Implement modular architectures where specific components (such as external tools)[44] handle different aspects of multi-step reasoning (for example, one module for peak identification, another for relative ordering).

10 Sensitivity to noise in image

The noise ablation analysis reveals significant differences in model robustness across tasks and models. While some models maintain relatively stable performance regardless of noise (Llama 3.2-90B on Electronic Structure), others exhibit threshold effects where performance collapses after certain noise levels (Claude 3.5 Sonnet on Electronic Structure). GPT-4o shows the steepest performance drop in all the tasks, while Llama 3.2-90B degrades more gradually. The almost stable performance of XRD might be due to distinctive peak patterns at specific positions, which make them recognizable even when additional noise is introduced.

11 Methodology for creating MCQ options

The options for all manually curated tasks are created with distractors that challenge easy eliminations. Table in Supplementary Data explains in detail how options were created for each of the tasks.

References

1. Darvish, K. *et al.* ORGANA: A robotic assistant for automated chemistry experimentation and characterization. *Matter* **8,** 101897. ISSN: 2590-2385. http://dx.doi.org/10.1016/j.matt.2024.10.015 (Feb. 2025).
2. Ruan, Y. *et al.* An automatic end-to-end chemical synthesis development platform powered by large language models. *Nature Communications* **15.** ISSN: 2041-1723. http://dx.doi.org/10.1038/s41467-024-54457-x (Nov. 2024).
3. Song, T. *et al.* A Multiagent-Driven Robotic AI Chemist Enabling Autonomous Chemical Research On Demand. *Journal of the American Chemical Society.* ISSN: 1520-5126. http://dx.doi.org/10.1021/jacs.4c17738 (Mar. 2025).
4. Liu, H., Li, C., Wu, Q. & Lee, Y. J. Visual Instruction Tuning. *arXiv preprint arXiv:2304.08485* (2023).
5. OpenAI. *Hello GPT-4o* https://openai.com/index/hello-gpt-4o/. May 13, 2024.
6. Team, G. *et al.* Gemini 1.5: Unlocking multimodal understanding across millions of tokens of context. *arXiv preprint arXiv:2403.05530* (2024).
7. Anthropic. *Claude 3.5 Sonnet* https://www.anthropic.com/news/claude-3-5-sonnet. June 21, 2024.
8. Jeong, D. P., Garg, S., Lipton, Z. C. & Oberst, M. Medical Adaptation of Large Language and Vision-Language Models: Are We Making Progress? *arXiv preprint arXiv:2411.04118* (2024).
9. Gupta, H. *et al.* Polymath: A Challenging Multi-modal Mathematical Reasoning Benchmark. *arXiv preprint arXiv:2410.14702* (2024).
10. Zhang, R. *et al.* MathVerse: Does Your Multi-modal LLM Truly See the Diagrams in Visual Math Problems? *arXiv preprint arXiv:2403.14624* (2024).
11. Zou, C. *et al.* DynaMath: A Dynamic Visual Benchmark for Evaluating Mathematical Reasoning Robustness of Vision Language Models. *arXiv preprint arXiv:2411.00836* (2024).
12. Li, Z. *et al.* MMSci: A Dataset for Graduate-Level Multi-Discipline Multimodal Scientific Understanding. *arXiv preprint arXiv:2407.04903* (2024).
13. Liang, Z. *et al. SceMQA: A Scientific College Entrance Level Multimodal Question Answering Benchmark* in *Proceedings of the 62nd Annual Meeting of the Association for Computational Linguistics (Volume 2: Short Papers)* (eds Ku, L.-W., Martins, A. & Srikumar, V.) (Association for Computational Linguistics, Bangkok, Thailand, Aug. 2024), 109–119. https://aclanthology.org/2024.acl-short.11.
14. Yue, X. *et al.* MMMU: A Massive Multi-discipline Multimodal Understanding and Reasoning Benchmark for Expert AGI. *arXiv preprint arXiv:2311.16502* (2024).

15. Chia, Y. K., Han, V. T. Y., Ghosal, D., Bing, L. & Poria, S. PuzzleVQA: Diagnosing Multimodal Reasoning Challenges of Language Models with Abstract Visual Patterns. *arXiv preprint arXiv:2403.13315* (2024).
16. Shao, H. *et al.* Visual CoT: Advancing Multi-Modal Language Models with a Comprehensive Dataset and Benchmark for Chain-of-Thought Reasoning. *arXiv preprint arXiv:2403.16999* (2024).
17. Roberts, J. S. *et al.* Image2Struct: Benchmarking Structure Extraction for Vision-Language Models. *arXiv preprint arXiv:2410.22456* (2024).
18. Zhang, D. *et al.* MM-LLMs: Recent Advances in MultiModal Large Language Models. *arXiv preprint arXiv:2401.13601* (2024).
19. Cheng, K. *et al.* Vision-Language Models Can Self-Improve Reasoning via Reflection. *arXiv preprint arXiv:2411.00855* (2024).
20. Laurent, J. M. *et al.* LAB-Bench: Measuring Capabilities of Language Models for Biology Research. *arXiv preprint arXiv:2407.10362* (2024).
21. Zhang, D. *et al.* ChemLLM: A Chemical Large Language Model. *arXiv preprint arXiv:2402.06852* (2024).
22. Roberts, J., Han, K., Houlsby, N. & Albanie, S. SciFIBench: Benchmarking Large Multimodal Models for Scientific Figure Interpretation. *arXiv preprint arXiv:2405.08807* (2024).
23. Khalighinejad, G. *et al.* MatViX: Multimodal Information Extraction from Visually Rich Articles. *arXiv preprint arXiv:2410.20494* (2024).
24. Schrödinger, LLC. *The PyMOL Molecular Graphics System, Version 1.8* Nov. 2015.
25. Rajan, K., Zielesny, A. & Steinbeck, C. DECIMER 1.0: deep learning for chemical image recognition using transformers. *Journal of Cheminformatics* **13.** ISSN: 1758-2946. `http://dx.doi.org/10.1186/s13321-021-00538-8` (Aug. 2021).
26. Rajan, K., Brinkhaus, H. O., Zielesny, A. & Steinbeck, C. Advancements in hand-drawn chemical structure recognition through an enhanced DECIMER architecture. *Journal of Cheminformatics* **16.** ISSN: 1758-2946. `http://dx.doi.org/10.1186/s13321-024-00872-7` (July 2024).
27. Cui, J., Chiang, W.-L., Stoica, I. & Hsieh, C.-J. *OR-Bench: An Over-Refusal Benchmark for Large Language Models* 2024. arXiv: `2405.20947 [cs.CL]`. `https://arxiv.org/abs/2405.20947`.
28. Chen, B., Zhang, Z., Langrené, N. & Zhu, S. Unleashing the potential of prompt engineering in Large Language Models: a comprehensive review. *arXiv preprint arXiv:2310.14735* (2024).
29. Chang, K. *et al.* Efficient Prompting Methods for Large Language Models: A Survey. *arXiv preprint arXiv:2404.01077* (2024).

30. Xu, Z. *et al.* Compress, Then Prompt: Improving Accuracy-Efficiency Trade-off of LLM Inference with Transferable Prompt. *arXiv preprint arXiv:2305.11186* (2023).

31. Amatriain, X. Prompt Design and Engineering: Introduction and Advanced Methods. *arXiv preprint arXiv:2401.14423.* arXiv: `2401.14423 [cs.SE]`. `https://arxiv.org/abs/2401.14423` (2024).

32. Han, S. J., Ransom, K. J., Perfors, A. & Kemp, C. Inductive reasoning in humans and large language models. *Cognitive Systems Research* **83,** 101155. ISSN: 1389-0417. `http://dx.doi.org/10.1016/j.cogsys.2023.101155` (Jan. 2024).

33. Zahan, N., Burckhardt, P., Lysenko, M., Aboukhadijeh, F. & Williams, L. Leveraging Large Language Models to Detect npm Malicious Packages. *arXiv preprint arXiv:2403.12196* (2025).

34. White, A. D. The future of chemistry is language. *Nature Reviews Chemistry* **7,** 457–458. ISSN: 2397-3358. `http://dx.doi.org/10.1038/s41570-023-00502-0` (May 2023).

35. Shen, J., Dudley, J. J., Zheng, J., Byrne, B. & Kristensson, P. O. Promptor: A Conversational and Autonomous Prompt Generation Agent for Intelligent Text Entry Techniques. *arXiv preprint arXiv:2310.08101* (2023).

36. Cao, R. *et al.* Spider2-V: How Far Are Multimodal Agents From Automating Data Science and Engineering Workflows? *arXiv preprint arXiv:2407.10956* (2024).

37. Wei, J. *et al.* Chain-of-Thought Prompting Elicits Reasoning in Large Language Models. *arXiv preprint arXiv:2201.11903* (2023).

38. Weng, Y. *et al.* Large Language Models are Better Reasoners with Self-Verification. *arXiv preprint arXiv:2212.09561* (2023).

39. Abid, A. *et al.* Gradio: Hassle-Free Sharing and Testing of ML Models in the Wild. *arXiv preprint arXiv:1906.02569* (2019).

40. Lab of Kevin Jablonka at Uni Jena. *MaCBench-Results (Revision 0551909)* 2025. `https://huggingface.co/datasets/jablonkagroup/MaCBench-Results`.

41. Snell, C., Lee, J., Xu, K. & Kumar, A. Scaling LLM Test-Time Compute Optimally can be More Effective than Scaling Model Parameters. *arXiv preprint arXiv: 2408.03314* (2024).

42. Muennighoff, N. *et al.* s1: Simple test-time scaling. *arXiv preprint arXiv: 2501.19393* (2025).

43. DeepSeek-AI *et al.* DeepSeek-R1: Incentivizing Reasoning Capability in LLMs via Reinforcement Learning. *arXiv preprint arXiv: 2501.12948* (2025).

44. Ramos, M. C., Collison, C. J. & White, A. D. A Review of Large Language Models and Autonomous Agents in Chemistry. *Chemical Science* (2025).

Acronyms

LLM large language model.

MAE mean absolute error.

SMILES simplified molecular input line-entry system.

VLLM Vision Large Language Model.
